# Transcriptomic Profiles and Functional Correlates of Cancer-Related Fatigue: A Cross-Sectional Study in Women Undergoing Cancer Treatment

**DOI:** 10.1155/ecc/1092518

**Published:** 2025-06-05

**Authors:** Amber S. Kleckner, Evelina Mocci, Carin L. Clingan, Shari M. Youngblood, Paula Y. Rosenblatt, Katherine H. R. Tkaczuk, Ian R. Kleckner, Susan G. Dorsey

**Affiliations:** 1 Department of Pain and Translational Symptom Science, University of Maryland School of Nursing, Baltimore, Maryland, USA; 2 University of Maryland Marlene and Stewart Greenebaum Comprehensive Cancer Center, Baltimore, Maryland, USA; 3 Institute of Genome Sciences, University of Maryland School of Medicine, Baltimore, Maryland, USA; 4 Department of Integrative and Functional Nutrition, Saybrook University, Pasadena, California, USA; 5 Department of Medicine, University of Maryland School of Medicine, Baltimore, Maryland, USA

## Abstract

**Background and Objectives:**

Cancer-related fatigue is a multifactorial condition that affects most people undergoing chemotherapy. To elucidate potential biological underpinnings of fatigue, this study tested correlations between patient-reported fatigue and (1) functional measures and (2) transcriptomics of whole blood.

**Methods:**

Women undergoing chemotherapy were recruited to a cross-sectional study. Participants reported subjective fatigue on the Functional Assessment for Chronic Illness Therapy-Fatigue (FACIT-F) and Brief Fatigue Inventory (BFI) questionnaires. Participants completed upper- and lower-body functional assessments as an objective fatigability measure. Fasted blood samples were analyzed for complete blood counts (CBCs) to quantify cell type, and RNA-Seq on whole blood was investigated for a distinct transcriptional signature in patients with high vs. low fatigue. Principal component analysis revealed that transcriptomic profiles clustered based on the neutrophil level, lymphocyte level, and several other clinical factors, which were accounted for when assessing differentially expressed genes.

**Results:**

Participants had breast (*n* = 29) or uterine (*n* = 1) cancer, were 53.4 ± 13.5 years old, and identified as Black/African American (56%) or White (44%). Hand grip strength, static fatigue index, and sit-to-stand assessments were not associated with FACIT-F fatigue subscale responses. From RNA-Seq data, higher fatigue was associated with fewer *SH3RF1* transcripts (*p* = 4.1*e*-3) and more *CAPRIN2* transcripts (*p* = 8.2*e*-3). Unbiased gene ontology/pathway analyses revealed perturbed biological processes in mitochondrial function, chiefly aerobic respiration (normalized effect size [ES] = −2.1), electron transport chain (ES = −1.9), generation of precursor metabolites and energy (ES = −1.8), and fatty acid oxidation (ES = −2.0), which tended to be downregulated among participants with more fatigue. Cellular components analyses consistently showed downregulation of mitochondrial proteins among those with higher fatigue (ESs = −1.7–−2.2). Future studies should investigate dietary, physical activity, and/or pharmaceutical interventions to optimize the efficiency of mitochondrial energy production during treatment to mitigate fatigue.

## 1. Introduction

Cancer-related fatigue affects at least 30%–90% of the patients undergoing chemotherapy, depending on the type of cancer, type of treatment, and method of fatigue measurement [1–3]. It is not relieved by sleep or rest, and its severity can greatly hinder one’s ability to perform activities of daily living. Moreover, fatigue tends to cluster with other debilitating symptoms such as depression and cognitive impairment as well as decreased quality of life [4, 5]. The mechanisms behind the etiology and pathophysiology of cancer-related fatigue are related in part to inflammation, hypothalamic–pituitary–adrenal (HPA) activation dysfunction, metabolic and/or endocrine dysregulation, circadian disruption, or other mechanisms but are largely not understood, thereby thwarting the development of effective preventative strategies and treatments [6–8]. While fatigue can stem from the tumor metabolism, psychological or social stress of a cancer diagnosis, biological effects of chemotherapy and other treatments, and other sources, we cannot specifically attribute it to a specific cause and, therefore, we refer to fatigue herein as “cancer-related fatigue.”

Transcriptomics is a useful approach to capture a snapshot of overall biology and physiology of a cell or tissue [9]. Quantification of RNA transcripts from whole blood (~15,000 genes) results in a profile that can be compared across circumstances or populations. By assessing differences in profiles between two populations or time points, one can compare these differences to investigate the biological pathways that might be differentially up- or downregulated [9]. The transcriptomic profiles identified in this study can have three major impacts: (1) to elucidate underlying mechanisms of cancer-related fatigue, (2) to develop and test novel therapeutic interventions based on these mechanisms [8], and (3) to develop biomarkers to predict, monitor, and subtype cancer-related fatigue [10].

There have been several studies that have specifically probed gene expression in the context of cancer-related fatigue. Specifically, there have been several longitudinal (e.g., [11–14]) and cross-sectional (e.g., [15–24]) studies and at least one randomized controlled trial that looked at fish vs. soybean oil for treatment of cancer-related fatigue (Peppone et al. [25, 26]); most were performed posttreatment. Collectively, these studies highlight the strong association between fatigue and inflammation (e.g., [12, 23]) and reveal other underlying mechanisms of fatigue, including impaired mitochondrial function (e.g., [13]) and dysregulated circadian rhythms (e.g., [21, 22]). Notably, Kober et al. examined transcriptomic profiles of 717 patients undergoing chemotherapy with vs. without fatigue, about half with RNA-Seq and about half with microarrays [23]. The data corroborated that inflammatory pathways are perturbed in people experiencing fatigue and revealed distinctions between morning and evening fatigue. However, blood samples in that study were not acquired in the fasting state, precluding analysis of nutrient metabolism pathways. In addition, the idea to test for correlations between transcriptomic profiles and objective measures of fatigue (e.g., muscle fatigability) is understudied and was not considered in the above referenced studies.

Herein, we selectively recruited 30 women with a range of fatigue experiences toward the middle or end of their chemotherapy regimen. We focused on women because females vs. males tend to report higher fatigue [3], and we recruited from breast, gynecological, and gastrointestinal cancer clinics because many of these cancers are curable with chemotherapeutic agents that cause fatigue, such as taxane, anthracycline, and/or platinum agents. We used bulk RNA-Seq from whole blood to examine transcriptomic profiles and hypothesized that genes related to metabolic pathways, inflammatory pathways, and hormonal pathways may be perturbed among those with high vs. low fatigue. We predict that better understanding of the differentially expressed genes (DEGs) and perturbed pathways among those with high vs. low fatigue can be leveraged in existing and novel interventions to address fatigue in a precision-medicine approach [27], as well as to lead to biomarkers to predict, monitor, and subtype fatigue during chemotherapy treatment.

## 2. Materials and Methods

### 2.1. Study Design and Participants.

The Transcriptomic Profiles of Cancer-Related Fatigue Cross-Sectional (TRIXIE) study was conducted at the University of Maryland Marlene and Stewart Greenebaum Comprehensive Cancer Center in Baltimore, Maryland, between October 2022 and December 2023. The primary aim was to investigate the transcriptomic profile of patients with cancer experiencing high vs. low levels of fatigue during chemotherapy treatment.

Participants were eligible if they had a diagnosis of breast, gastrointestinal, or gynecological cancer, stage 0, 1, 2, or 3; were assigned female sex at birth; had at least 4 weeks and 2 cycles of chemotherapy and at least one scheduled session remaining; could communicate in English; and were at least 18 years old. This study was approved by the University of Maryland Institutional Review Board (HP-00100268). All participants provided written informed consent.

### 2.2. Demographics, Fatigue Measures, and Functional Assessments.

Demographics were obtained from a structured questionnaire and clinical characteristics from medical records.

Questionnaires were completed either the same day as the blood draw and functional assessments or within the previous 5 days. Patient-reported fatigue was assessed using the Functional Assessment of Chronic Illness Therapy-Fatigue (FACIT-F) [28], Brief Fatigue Inventory (BFI) [29], and a Symptom Inventory. The FACIT-F is a 40-item, validated measure that comprises five subscales: physical wellbeing, social wellbeing, emotional wellbeing, functional wellbeing, and a fatigue subscale [28]. Participants responded with how true various statements were over the last 7 days such as “I feel fatigued” and “I have to limit my social activities because I am tired” with five response choices ranging from 0, “Not at all,” to 4, “Very much.” Scoring yields five subscale scores, a total score, a general quality of life score (Functional Assessment of Cancer Therapy-General [FACT-G]; physical + social + emotional + functional wellbeing), and a trial outcome index (TOI; physical wellbeing + functional wellbeing + fatigue subscale). A higher score indicates higher wellbeing/quality of life or less fatigue. The BFI is a 10-item questionnaire that assesses fatigue now, usual, and worst fatigue in the last 24 h and how much fatigue has interfered with general activity, mood, etc. [29]. The item scales range from 0, “No fatigue” or “Does not interfere,” to 10, “As bad as you can imagine” or “Completely interferes.” The average of all the items yields a global fatigue score with a higher score indicating worse fatigue. Cronbach alpha reliability ranges from 0.82 to 0.97 [29]. Lastly, there were four questions related to fatigue as part of a Symptom Inventory—fatigue, weakness, drowsiness, and sleep problems. Participants rated the symptoms at their worst in the past seven days from 0, “Not present,” to 10, “As bad as you can imagine.”

Functional assessments were performed the same day as the blood draw after a snack was offered. Grip strength and muscle fatigability were assessed using handgrip dynamometry (handheld Hydraulic Hand Dynamometer, Model 5030J1, Jamar Technologies, Hatfield, PA, USA). Maximal voluntary isometric contraction (MVIC) was assessed on both the left and right arms using the protocol described in Mustian et al. [30]. In brief, the participant stood with their elbow joint at a constant 90° angle and the instrument was positioned so the palm fit comfortably in the rear of the instrument and the fingers curled around the adjustable grip. The participants were asked to squeeze as hard as possible and hold for 5 s. Several practice squeezes were performed, after which three trials were performed with each hand, alternating, with at least 30 s between trials. The measurement from the peak-hold needle was recorded. The average of the three trials was used in the analysis.

At least 1 min after the MVIC test, muscle fatigability was assessed via the static fatigue index using the same dynamometer [31]. For this trial, the participant squeezed the dynamometer maximally and held it for 30 s. Maximal force was recorded, as well as force at 5, 10, 15, 20, 25, and 30 s. The area under the curve was calculated (equal to AUC2), as well as the maximum possible AUC based on the maximal force (AUC_max_). Static fatigue index was calculated as 100% × (1 − AUC2/AUC_max_) (Supporting Figure 1). A greater score indicates less fatigability.

The 30-s chair stand test is a measure of leg endurance and muscular endurance [32]. We measured the number of times the participant could rise from a sitting position in a standard chair in 30 s without assistance, holding onto the chair or other objects, or using their arms.

### 2.3. Blood Collection and Complete Blood Count (CBC).

A fasted blood draw was performed in conjunction with clinical labs approximately 1–2 h before the participant’s chemotherapy appointment. The clinical labs included a CBC of hemoglobin, red blood cells (RBCs), white blood cells (WBCs), platelets, neutrophils, lymphocytes, and monocytes; these values were obtained from the medical record. For RNA-Seq, venous blood (3 mL) was collected into a Tempus tube (ThemoFisher Scientific, Waltham, MA, USA) containing 6 mL of stabilizing reagent, which inactivates cellular RNases and selectively precipitates RNA. The tubes were placed at −80°C within 2 h and stored there until processing.

### 2.4. RNA-Seq.

RNA was extracted and sequenced at the Genomics Core Facility, University of Maryland School of Medicine (UMSOM), using the Tempus Spin RNA Isolation Reagent Kit (ThermoFisher). Extracted RNA was run on Bioanalyzer gels to obtain the RNA integrity number (RIN; all were ≥ 7.4 on the quality scale).

At Maryland Genomics, part of the Institute of Genome Sciences, libraries were prepared from 25 ng of RNA using the TruSeq RNA Sample Prep kit (Illumina, San Diego, CA, USA) according to the manufacturer’s instructions, with an additional PCR cycle. Samples were sequenced on an Illumina NovaSeq6000 with a 150 base pair (bp) paired-end read configuration. Sequences were checked for overall quality using FastQC [33], and low-quality reads, duplicates, and adapter surplus were trimmed using Trimmomatic [34]. Filtered reads were aligned to the human reference Ensembl Homo sapiens.GRCm38.96 using HiSat (Version HISAT2–2.0.4) [35], and the number of reads by gene was determined using HTSeq [36]. Overall, 29/30 samples met quality control criteria and were used in further analyses.

### 2.5. Statistical Analysis.

Demographics, clinical characteristics, fatigue, and functional measures were compared between the two groups using a *t*-test allowing unequal variances (Microsoft Excel, Redmond, WA, USA, or JMP, SAS Institute, Cary, NC, USA).

For DGE analysis, gene counts were normalized to the log_2_ scale, and genes with low mean normalized counts were removed from the analysis. Normalized and filtered gene counts were utilized in the principal component analysis (PCA), which enabled the identification of any outliers and clusters of samples by demographic and/or clinical data. The variables that showed associations with the first two principal components (PCs) were used as covariates in the downstream DGE analysis. To detect genes associated with fatigue, we used a dual approach; first, following the criteria applied by Eek et al. [37], we set a fatigue cutoff of 30 on the FACIT-F fatigue subscale to dichotomize samples into high (> 30, *n* = 14) and low (≤ 30, *n* = 15) fatigue and compared their gene expression. In parallel, we were interested in evaluating gene expression changes by changes in fatigue score units. While using fatigue as a continuous variable is less useful for clinical applications, it is statistically more powerful and avoids the establishment of arbitrary cutoffs that could alter the biological interpretation of the analysis results. Both analyses were conducted using DESeq2, which models gene expression data using the negative binomial distribution [38]. We applied the likelihood ratio test and computed *p* values as the difference in deviance between the full models (including cancer stage, WBC, neutrophils, lymphocytes, RBC, platelet, and fatigue score or group [high/low]) and reduced models (including cancer stage, WBC, neutrophils, lymphocytes, RBC, and platelets). We considered DEGs statistically significant if their Benjamini–Hochberg adjusted *p* value was equal to or lower than 0.1.

We utilized CIBERSORTx [39–41] to infer whole blood cell-type fractions from RNA-seq data. To this aim, we first normalized the gene counts as counts per million (CPM) and used a signature gene expression matrix (LM22) implemented in the software that cell-type specific markers. To confirm the accuracy of the fractions of each blood cell type estimated using the CIBERSORTx, we assessed for correlations for neutrophil and monocyte concentrations between those estimated with CIBERSORTx and those that were measured clinically. We found strong correlations (neutrophils *R*^2^ = 0.85 and monocytes *R*^2^ = 0.56, Supporting Figure 2).

For pathway analysis, we ranked all genes analyzed for differential expression by the product of their log_2_fold-change (log_2_FC) and *p* values. We tested them for enrichment in biological processes/pathways using Gene Ontology (GO)’s biological processes functionally related to cancer-related fatigue. As gene sets for the analysis, we used the collection gene set C5 (Version 2023) that derives from the GO resource [42] and contains biological processes, cellular components, and molecular functions in addition to gene sets derived from the Human Phenotype Ontology (HPO) [43]. The enrichment score (ES) and its significance were computed with the multilevel algorithm implemented in the package fast gene set enrichment analysis (fgsea) [44], which performs permutations to adjust for multiple testing. In addition to the *p* value and adjusted *p* value, fgsea output includes an ES whose sign (positive or negative) indicates whether the larger part of the genes in the considered gene set is up- or downregulated. In addition, fgsea highlights the leading genes, which have the largest difference in expression and give the strongest contribution to the gene set. Subsequently, we conducted sensitivity analysis using the “leave one out” method [45]. We considered the DEG finding robust when significance was retained for > 90% of the trials.

## 3. Results

### 3.1. Sample Description.

A total of 68 women were approached; 38 did not enroll (18 declined and 20 were not eligible [10 had stage 4 disease and/or metastases, 2 were receiving chemotherapy at a different hospital, 4 did not speak English, and 4 did not have any more chemotherapy scheduled]) and 30 women enrolled between November 2022 and November 2023 (Table 1). Participants were 53.4 ± 13.5 years old. Approximately half identified as Black and/or African American and half identified as White. Participants were on average overweight (BMI: 25.8 ± 7.2 kg/m^2^), consistent with the American population. Most (96.7%) had breast cancer and one had uterine cancer. Participants were on a variety of chemotherapy regimens.

### 3.2. Fatigue and Functional Assessments.

Participants experienced a wide range of self-reported fatigue as measured by the FACIT-F, BFI, and single-item questions (Table 2). Participants were dichotomized according to fatigue level (≤ 30 for low vs. > 30 for high on the FACIT-F fatigue subscale [37]) for some subsequent analyses; there were no statistically significant differences between these two groups in regard to demographics or clinical characteristics (Table 1). Interestingly, while those in the “low fatigue” group had less fatigue and/or greater wellbeing on all scales, statistical significance was reached only for physical wellbeing, functional wellbeing, the BFI total score, and the single-item questions for fatigue, weakness, and drowsiness, and not social wellbeing, emotional wellbeing, or sleep problems. In regard to physical assessments, those with low fatigue exhibited higher grip strength and lower fatigability, though differences did not reach statistical significance.

### 3.3. Transcriptome Analyses.

We obtained an average of > 106 million bp paired ends reads per sample, and 96.62% of them matched properly to the reference, guaranteeing an average of 10× coverage of the transcriptome (Supporting Table 1). Of the mapped reads, 93.07% aligned to exons, 5.94% to introns, and 0.99% to intergenic regions.

While we did not observe any outliers, PCA analysis enabled us to detect significant clustering of the first two components (PC1 and PC2) of expressions of transcripts by cancer stage, overall WBC counts, and singular neutrophils and lymphocyte counts. In addition, PC1 was significantly correlated with RBCs and platelet counts (Supporting Figure 3). These variables were used as covariates in the DEG analyses.

**Figure 1:**
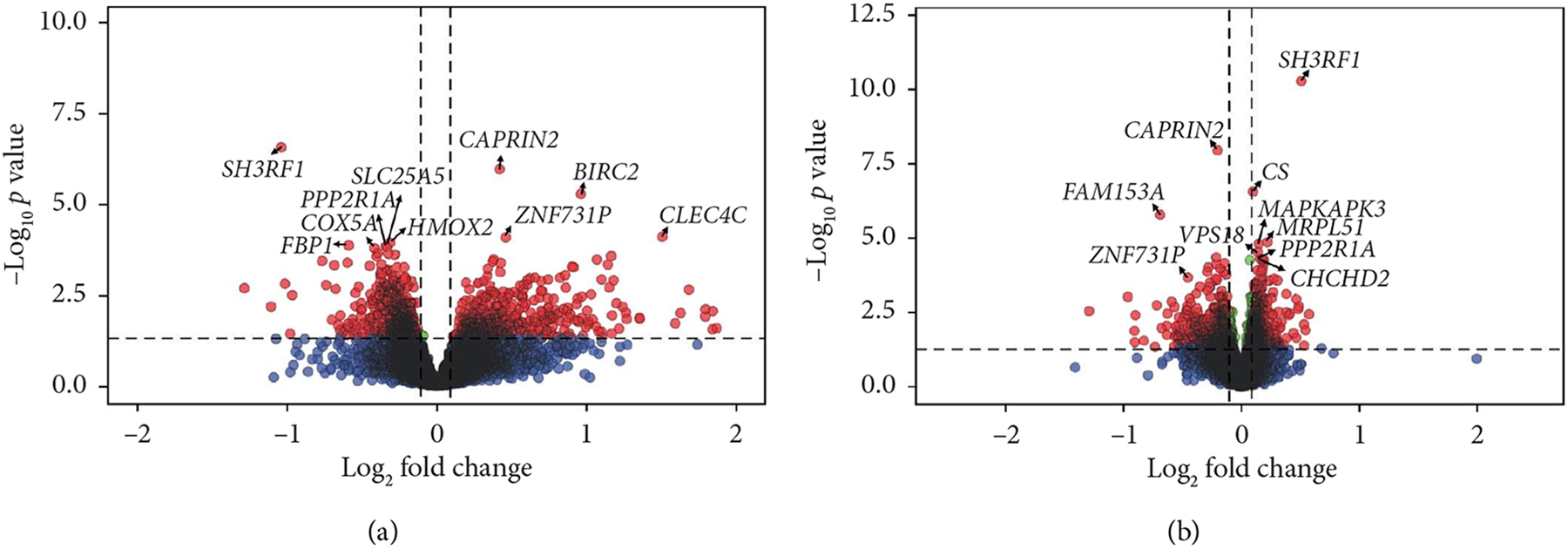
Enhanced volcano plots illustrating statistical significance (*p* value) versus magnitude of change (fold change) for differentially expressed genes. (a) Participants were dichotomized into those who reported high fatigue (> 30 points on the Functional Assessment of Chronic Illness Therapy-Fatigue (FACIT-F), fatigue subscale, *n* = 14) vs. low fatigue (≤ 30 points, *n* = 15). (b) FACIT-F fatigue score was used as a continuous variable. For this scale, a higher score reflects a greater quality of life and less fatigue. For example, both of these plots illustrate that *SH3RF1* is downregulated with more fatigue. Black = not significant, blue = log_2_ fold-change is significant, green = − log_10_ *p* value is significant, and red = − log_10_ *p* value and log_2_ fold-change are significant. There was a total of 9551 variables in each plot.

Comparison of gene expression between the high and low fatigue groups highlighted three DEGs: SH3 domain containing ring finger 1 (*SH3RF1*) alias *POSH* (Log_2_FC = −1.04, FDR = 4.08*e* – 03), caprin family member 2 (*CAPRIN2*, Log_2_FC = 0.42, FDR = 8.23*e* – 03), and baculoviral IAP repeat containing 2 (*BIRC2*, Log_2_FC = 0.97, FDR = 2.42*e* – 02) (Figure 1, Supporting Table 2, Supporting Figure 4). With higher fatigue coded as a continuous score, *SH3RF1* again was found to be significantly downregulated and *CAPRIN2* upregulated with higher fatigue, and there was significant perturbation of eight other genes: citrate synthase (*CS*), family with sequence similarity 153 member A (*FAM153A*), mitochondrial 54S ribosomal protein MRPL51 (*MRPL51*), MAPK activated protein kinase 3 (*MAPKAPK3*), VPS18 core subunit of CORVET and HOPS complexes (*VPS18*), zinc finger protein 731 (*ZNF731P*), protein phosphatase 2 scaffold subunit alpha (*PPP2R1A*), and coiled-coil-helix-coiled-coil-helix domain containing 2 (*CHCHD*2) (Supporting Table 3). A sensitivity analysis was performed using the “leave one out” method [45]; key genes identified using linear regression were confirmed to be robust to potential outliers (i.e., *SH3RF1*, *CAPRIN2*, and *BIRC2*, Supporting Table 4).

All genes were then ranked in decreasing order by the product of the log_2_FC and significance and tested their enrichment in GO (biological processes, cellular components, and molecular functions) and HPO gene sets using fgsea [43] (Table 3). Twenty-four biological processes were significantly enriched, related to mitochondrial function (e.g., aerobic respiration, generation of precursor metabolites, and electron transport chain), ATP synthesis, and fatty acid β-oxidation. In addition, DEGs were primarily enriched in mitochondria and ribosomal cellular components and molecular functions linked with mitochondrial protein complexes and oxidoreductase (Table 3). Interestingly, the ES was negative for all the GO and HPO terms, indicating that most genes included in the gene sets were downregulated in samples with high fatigue compared to low fatigue. Results of the enrichment analysis were similar when we analyzed fatigue as a continuous variable, but the ES increased with increasing fatigue score (Supporting Table 5).

## 4. Discussion

This study explored mechanisms underlying cancer-related fatigue via patient-reported outcomes, functional assessments, and blood-based transcriptomics among women actively undergoing chemotherapy for cancer. While cross-sectional assessments of function and fatigability did not correlate with subjective fatigue, changes in these objective assessments over time may be useful in future longitudinal studies. This is one of the first studies to characterize the transcriptome of patients with cancer experiencing high vs. low fatigue using fasted peripheral blood samples and controlling for blood cell type. Pathways involved in mitochondrial function and energy metabolism tended to be significantly downregulated in patients with higher vs. lower fatigue. These pathways elucidate metabolic deficits that may be modifiable with behavioral and pharmaceutical interventions and are consistent with known beneficial effects of exercise on mitochondrial function and fatigue [46, 47].

The gold standard for measuring cancer-related fatigue is patient report; while fatigue is a subjective experience by nature, a sensitive, specific, and validated biomarker or functional assessment would be useful to objectively assess, monitor, or subtype cancer-related fatigue. There are some measures that correlate with cancer-related fatigue, for example, muscle “fatigability,” as in during exercise, that complement subjective measures [48–50]. In addition, functional measures can provide indirect measures of ATP availability and efficiency of energy production. These measures are most useful when looking at changes in fatigability over time (with or without treatment or an intervention), due to people’s variability in baseline muscular fitness. In a 2021 meta-analysis of muscle strength and cancer-related fatigue [51], six of nine studies showed an inverse relationship between grip strength and fatigue (four studies with patients during treatment, one after treatment, and one receiving palliative care). The meta-analysis also concluded an inverse association between knee extensor strength and fatigue [51]. In our study, participants who reported lower fatigue exhibited slightly higher strength and lower fatigability, but the differences did not meet statistical significance (effect sizes: 0.2–0.4). We expect that these measures would be more sensitive to changes over time; in other words, people who experience large decreases in hand grip strength would experience higher levels of cancer- or treatment-induced fatigue.

We found two genes that met thresholds for statistical significance in both binary and continuous fatigue score analyses—*SH3RF1* (lower expression with higher fatigue) and *CAPRIN2* (higher expression with higher fatigue). *SH3RF1* is involved in protein sorting at the trans-Golgi network, and *CAPRIN2* is involved in mRNA transport. There is limited literature on the role of either *SH3RF1* or *CAPRIN2* in energy regulation and fatigue. However, higher expression of *SH3RF1* is related to increased “stemness” properties of breast cancer cells [52] and *CAPRIN2* may also play a role in carcinogenesis [53].

Transcriptomics pathway results highlight associations between mitochondrial dysfunction and fatigue and are consistent with others [7]. For example, in an analysis of 33 patients, most of whom had breast cancer, patient-reported fatigue was associated with reduced mitochondrial respiratory capacity in freshly isolated T cells [54]. Also, among a study of breast cancer survivors in early posttreatment survivorship, participants with a lower expression of genes from mitochondrial DNA had greater improvements in fatigue over the following 6 weeks, suggesting that mitochondrial function was compromised at enrollment [26]. In 2013, Saligan et al. revealed upregulation of α-synuclein with cancer-related fatigue during radiation; a protein involved in mitochondrial fusion and fission [14]. Hsiao et al. observed positive associations between fatigue and *IFI27* expression, a gene that expresses a protein associated with inflammation and mitochondrial dysfunction, as measured using qRT-PCR [55]. Consistently, herein, we observed a +3.66-fold change in *IFI27* among those with high vs. low fatigue (Supporting Table 2), but the results did not reach statistical significance. In a separate study, Hsiao et al. recruited 15 patients with nonmetastatic prostate cancer receiving external beam radiation therapy (EBRT) and collected peripheral whole blood samples at 7 time points. They observed that mitochondrial-related gene expression changed over time and that gene expression of several genes was associated with fatigue—some positively and some negatively correlated [13].

Many previous transcriptomic studies exhibited associations between fatigue and inflammation and immune responses. For example, Landmark-Hoyvik et al. found differential expression in B-cell-mediated inflammatory processes among cancer survivors [56]. Bower et al. observed upregulation of pro-inflammatory cytokines and chemokine signaling genes in fatigued breast cancer survivors and later noted elevated Type I IFN response genes associated with fatigue [12, 24]. Also, Black et al. linked fatigue in colorectal cancer survivors with upregulated adaptive immune system activity [18]. Kober et al. performed a large study (> 700 participants) among patients undergoing chemotherapy with a distinction between morning and evening fatigue [23]. Gene expression and pathway impact analysis were performed with peripheral blood samples using RNA-Seq (*n* = 357) and microarray (*n* = 360; independent samples). Consistent with their prior work [19, 21, 22], differences in the expression of inflammatory pathways were seen between people with vs. without fatigue, and some of these pathways were distinct between morning and evening fatigue. When we controlled for neutrophil and lymphocyte quantities, we no longer observed differences in transcript quantity of genes related to inflammation between those with high vs. low fatigue. Collectively, these data suggest that inflammation is highly associated with fatigue, but gene regulation of these WBCs may not be perturbed among those with high vs. low fatigue.

### 4.1. Strengths and Limitations.

This study has several strengths. First, it collected blood from fasted participants and accounted for WBC population within blood, which helps identify metabolic perturbations beyond those in the fed vs. fasted state and differences in populations of inflammatory cells. Second, it recruited a racially diverse population of women undergoing chemotherapy, increasing the generalizability of our results to populations who have been historically underrepresented in research. Third, we used state-of-the-art transcriptomic analysis techniques to identify potential biological and physiological targets to prevent, treat, monitor, and eventually subtype cancer-related fatigue.

However, this study has some limitations. It is a relatively small study (*n* = 29–30) and, therefore, was underpowered to see differences between high vs. fatigue groups in regard to functional assessments (ES values ranging 0.2–0.4). Also, the population was heterogenous concerning cancer subtypes, cancer stages, and chemotherapy regimens, which might have reduced our ability to resolve differences between groups; our small sample size precluded our ability to adjust for any of these variables. However, our population did not have advanced cancer, which is associated with cachexia, and the muscle wasting syndrome potentially has independent causes of energy dysregulation and fatigue [57]. We assessed and did not find differences between groups with high vs. low fatigue in regard to age, BMI, and other potential confounders, but it is possible that residual confounding exists. Indeed, obesity can be associated with more fatigue [58] and different metabolic transcriptomic profiles [59]. We were able to control for WBC type, which helps to control for inflammatory state. Also, this was a cross-sectional study; fatigue can fluctuate greatly over the course of treatment [12], and there are large differences in physiology between individuals; longitudinal studies have more statistical power to assess changes within people vs. differences across people. In our dichotomous comparisons between those with high vs. low fatigue, it is possible that there was misclassification, for example, if the day or time during which they completed the fatigue questionnaires was not representative of their global fatigue experience. In order to avoid misclassification, we administered several questionnaires that inquired about fatigue over several time periods (e.g., now, in the last 7 days) and confirmed that these values correlated. In addition, we modeled fatigue as a continuous variable, which is more nuanced in fatigue quantification. Hence, these results should be interpreted with prudence and tested for confirmation in independent studies.

### 4.2. Clinical and Research Implications.

Fatigue is a multifactorial experience of physical and mental lack of energy with elusive underlying mechanisms. This study confirms current clinical practice guidelines that fatigue should be discussed frequently between the patient and the provider in order to recognize and manage it as best as possible [60, 61]. Further research is needed to incorporate functional and/or objective measures of fatigue into the clinic to complete patient-reported outcomes and help quantify and characterize it. While patient report is the current gold standard to diagnose and track fatigue, people experiencing moderate–severe fatigue may have reduced focus and concentration, which may reduce the accuracy of questionnaire data, especially for mental fatigue. Functional data are useful, especially in assessing changes over time, because they reflect physical aspects of fatigue that may not be noticeable and, therefore, not reportable. In addition, objective biomarkers such as blood-, saliva-, or urine-based measures should continue to be explored [62, 63]. While direct targeting of mitochondrial function has not yet led to interventions with large effect sizes (e.g., [64]), current recommendations such as exercise [61] and nutrition [54] may work through these pathways and future research may be able to optimize it.

## 5. Conclusions

Herein, using RNA-Seq, we describe perturbed biological processes in mitochondrial function, chiefly cellular/aerobic respiration, electron transport chain, generation of precursor metabolites and energy, and fatty acid oxidation, which tended to be downregulated among participants with high fatigue. These transcriptomic profiles suggest the potential to manipulate mitochondrial energy metabolism upstream during cancer treatment to mitigate fatigue, for example, through optimizing dietary, physical activity, and/or pharmaceutical interventions.

## Supplementary Material

suppl Supporting Figure 1: Calculation of the Static Fatigue Index. Supporting Figure 2: Heatmap correlating patient-reported fatigue, functional measures, and cell types as measured using clinical CBCs and RNA-Seq plus CIBERSORTx. Supporting Figure 3: PCA was used to identify clusters regarding demographics, clinical characteristics, or cell populations in blood. Supporting Figure 4: Raw data for an example single gene (*SH3RF1*) expression vs. fatigue. Supporting Table 1: Statistical alignment. Supporting Table 2: Differential Gene Expression for High vs Low Fatigue. Supporting Table 3: Differential Gene Expression for fatigue as a continuous variable. Supporting Table 4: Sensitivity analysis using the “leave one out” method. Supporting Table 5: Pathway analysis with fatigue as a continuous variable.

Additional supporting information can be found online in the Supporting Information section. (Supporting Information)

## Figures and Tables

**Table 1:** Demographics and clinical characteristics (*n* = 30).

	All participants Mean ± SE or *n* (%) *n* = 30	Low fatigue Mean ± SE or *n* (%) *n* = 16	High fatigue Mean ± SE or *n* (%) *n* = 14	*p* value*
Age (years)	53.4 ± 13.5	55.8 ± 12.4	50.7 ± 14.7	0.318
Race				0.961
Black/African American	17 (56.7%)	9 (56.3%)	8 (57.1%)	
White	13 (43.3%)	7 (43.8%)	6 (42.9%)	
Ethnicity				0.257
Hispanic	1 (3.3%)	1 (6.3%)	0	
Non-Hispanic	29 (96.7%)	15 (93.8%)	14 (100%)	
Body mass index (kg/m^2^)	25.8 ± 7.2	25.4 ± 7.4	26.3 ± 7.3	0.761
Cancer site				0.257
Breast	29 (96.7%)	15 (93.8%)	14 (100%)	
Uterus	1 (3.3%)	1 (6.3%)	0	
Stage				0.267
1	8 (26.7%)	6 (37.4%)	2 (14.3%)	
2	16 (53.3%)	8 (50.0%)	8 (57.1%)	
3	6 (20.0%)	2 (12.5%)	4 (28.5%)	
Chemotherapy regimen^†^				0.614
TCHP	8 (26.7%)	4 (25.0%)	4 (28.5%)	
Dose-dense AC	6 (20.0%)	3 (18.8%)	3 (21.4%)	
CP-AC	6 (20.0%)	2 (12.5%)	4 (28.6%)	
TC	5 (16.7%)	4 (25.0%)	1 (7.1%)	
Other	5 (16.7%)	3 (18.8%)	2 (14.3%)	

*Note:* Participants were dichotomized into low and high fatigue groups from the functional assessment at chronic illness therapy-fatigue (FACIT-F) fatigue subscale (≤ 30 for low fatigue and > 30 for high fatigue).

* *t*-test or chi-squared test (with likelihood ratio test) to compare the difference between those with high vs. low fatigue.

† TCHP: docetaxel, carboplatin, trastuzumab, pertuzumab; AC: adriamycin (doxorubicin), cyclophosphamide; CP-AC: carboplatin, paclitaxel, pembrolizumab followed by adriamycin and cyclophosphamide; TC: docetaxel, cyclophosphamide.

**Table 2:** Functional measures and patient-reported fatigue among patients undergoing chemotherapy.

	All participants Average ± standard deviation	Low fatigue (*n* = 16) Average ± standard deviation	High fatigue (*n* = 14) Average ± standard deviation	Effect size* High fatigue minus low	*p* value^†^
FACIT-F^‡^ fatigue subscale^§^	31.0 ± 10.2	38.4 ± 7.1	22.5 ± 5.3	−1.6	**<** 0.001
FACIT-F physical wellbeing	18.0 ± 5.5	21.1 ± 3.9	14.4 ± 4.8	−1.2	**<** 0.001
FACIT-F social wellbeing	21.9 ± 5.9	23.2 ± 5.5	20.5 ± 6.2	−0.5	0.216
FACIT-F emotional wellbeing	18.3 ± 4.1	19.3 ± 3.5	17.2 ± 4.5	−0.5	0.175
FACIT-F functional wellbeing	17.5 ± 5.2	19.9 ± 4.7	14.9 ± 4.5	−1.0	0.006
FACIT-F trial outcome index (TOI)^‖^	66.5 ± 17.8	79.4 ± 13.3	51.8 ± 8.0	−1.6	< 0.001
FACIT-F FACT-G^¶^	75.8 ± 15.4	83.5 ± 13.6	67.0 ± 12.8	−l.1	< 0.001
FACIT-F total score	106.8 ± 23.4	121.9 ± 18.6	89.5 ± 14.8	−1.4	< 0.001
Brief fatigue inventory total score	4.1 ± 2.4	2.8 ± 2.3	5.5 ± 1.5	1.1	< 0.001
Symptom inventory: fatigue	5.3 ± 2.7	3.8 ± 2.7	7.0 ± 1.9	1.2	< 0.001
Symptom inventory: weakness	4.8 ± 2.9	3.6 ± 2.9	6.3 ± 14.8	0.9	0.007
Symptom inventory: drowsiness	3.3 ± 2.6	1.9 ± 1.9	4.9 ± 2.4	1.2	0.001
Symptom inventory: sleep problems	4.0 ± 3.3	3.9 ± 3.6	4.1 ± 3.1	0.1	0.829
Handgrip (kg force)^#^					
Right	23.9 ± 8.0	25.5 ± 7.4	22.0 ± 8.5	−0.4	0.246
Left	22.6 ± 7.2	24.1 ± 7.0	22.9 ± 7.3	−0.2	0.224
Overall	25.1 ± 7.6	26.6 ± 6.9	23.4 ± 8.2	−0.4	0.262
Static fatigue index (%)^Δ^					
Right	36.1 ± 11.4	35.2 ± 11.2	37.1 ± 12.0	0.2	0.658
Left	37.5 ± 12.4	36.5 ± 12.5	38.7 ± 12.6	0.2	0.636
Overall	36.8 ± 11.0	35.8 ± 10.9	37.9 ± 11.4	0.2	0.620
Sit-to-stand (no.)	12.3 ± 4.0	12.3 ± 3.8	12.4 ± 4.4	0.0	0.986

* Effect sizes were calculated as [Average (High Fatigue) − Average (Low Fatigue)]/[Standard deviation (All)].

† *p* values were calculated using a *t*-test between those with low vs. high fatigue assuming unequal variances.

‡ FACIT-F: Functional Assessment of Chronic Illness Therapy–Fatigue. A greater score indicates greater wellbeing (less fatigue).

§ Participants were dichotomized based on the fatigue subscale: ≤ 30 = low fatigue and > 30 = high fatigue.

‖ Trial Outcome Index: physical wellbeing + functional wellbeing + fatigue subscale.

¶ FACT-G: Functional Assessment of Cancer Therapy–General.

# A higher score indicates greater strength.

Δ A higher score indicates higher fatigability.

**Table 3:** Result of fast gene set enrichment analysis (fgsea) considering high vs. low fatigue differentially expressed genes ordered by decreasing product of log-fold change and *p* value.

Pathway	*p* value	Adjusted *p* value	log_2_error	Enrichment score	Normalized effect size	Size
GObp_aerobic_respiration	2.61E - 08	4.27E - 05	0.734	−0.528	−2.088	131
GObp_generation_of_precursor_metabolites_and_energy	2.18E - 08	4.27E - 05	0.734	−0.422	−1.836	315
GObp_cellular_respiration	7.69E - 07	5.18E - 04	0.659	−0.485	−1.945	154
GObp_oxidative_phosphorylation	1.12E - 06	6.51E - 04	0.644	−0.549	−2.04	95
GObp_ncrna_metabolic_process	2.34E - 06	1.19E - 03	0.627	−0.358	−1.599	458
GObp_electron_transport_chain	4.75E - 06	1.94E - 03	0.611	−0.501	−1.927	112
GObp_atp_synthesis_coupled_electron_transport	7.21E - 06	2.37E - 03	0.611	−0.578	−2.018	66
GObp_proton_transmembrane_transport	6.98E - 06	2.37E - 03	0.611	−0.564	−2.041	78
GObp_rrna_metabolic_process	7.27E - 06	2.37E - 03	0.611	−0.426	−1.764	209
GObp_fatty_acid_beta_oxidation	1.50E - 05	4.22E - 03	0.593	−0.611	−2.022	50
GObp_respiratory_electron_transport_chain	1.64E - 05	4.46E - 03	0.576	−0.548	−1.982	77
GObp_ribonucleoprotein_complex_biogenesis	1.88E - 05	4.81E - 03	0.576	−0.364	−1.604	371
GObp_proton_motive_force_driven_atp_synthesis	2.62E - 05	5.93E - 03	0.576	−0.590	−1.961	52
GObp_rna_capping	2.57E - 05	5.93E - 03	0.576	−0.687	−2.044	30
GObp_localization_within_membrane	2.87E - 05	6.33E - 03	0.576	−0.350	−1.558	422
GObp_ribosome_biogenesis	3.12E - 05	6.63E - 03	0.557	−0.392	−1.661	248
GObp_ncrna_processing	4.07E - 05	7.91E - 03	0.557	−0.364	−1.592	327
GObp_monocarboxylic_acid_metabolic_process	4.34E - 05	8.06E - 03	0.557	−0.365	−1.599	334
GObp_nucleobase_containing_small_molecule_metabolic_process	4.28E - 05	8.06E - 03	0.557	−0.355	−1.561	365
GObp_spliceosomal_snrnp_assembly	4.72E - 05	8.48E - 03	0.557	−0.673	−2.045	33
GObp_lipid_oxidation	5.03E - 05	8.62E - 03	0.557	−0.543	−1.92	69
GObp_atp_biosynthetic_process	5.50E - 05	8.98E - 03	0.557	−0.534	−1.884	68
GObp_energy_derivation_by_oxidation_of_organic_compounds	6.28E - 05	9.32E - 03	0.538	−0.413	−1.71	201
GObp_ribosomal_large_subunit_biogenesis	6.60E - 05	9.46E - 03	0.538	−0.570	−1.94	55
GOcc_mitochondrial_protein_containing_complex	1.86E - 12	1.52E - 08	0.899	−0.541	−2.239	202
GOcc_organelle_inner_membrane	1.56E - 09	6.36E - 06	0.788	−0.429	−1.885	349
GOcc_inner_mitochondrial_membrane_protein_complex	3.27E - 09	8.91E - 06	0.775	−0.595	−2.248	103
GOcc_mitochondrial_matrix	6.51E - 07	4.83E - 04	0.659	−0.396	−1.74	345
GOcc_respirasome	8.43E - 06	2.65E - 03	0.593	−0.597	−2.079	63
GOcc_endoplasmic_reticuIum_protein_containing_complex	1.08E - 05	3.25E - 03	0.593	−0.517	−1.937	98
GOcc_u2_type_catalytic_step_2_spliceosome	2.21E - 05	5.47E - 03	0.576	−0.710	−2.085	28
GOcc_u2_type_spliceosomal_complex	3.51E - 05	7.16E - 03	0.557	−0.519	−1.891	83
GOcc_precatalytic_spliceosome	5.37E - 05	8.95E - 03	0.557	−0.610	−1.987	46
GOcc_atpase_complex	5.91E - 05	9.32E - 03	0.557	−0.483	−1.846	109
GOcc_large_ribosomal_subunit	6.17E - 05	9.32E - 03	0.538	−0.516	−1.875	81
GOcc_sm_like_protein_family_complex	6.07E - 05	9.32E - 03	0.557	−0.537	−1.918	73
GOmf_oxidoreductase_activity	2.40E - 07	2.45E - 04	0.675	−0.388	−1.713	389
GOmf_proton_transmembrane_transporter_activity	3.66E - 06	1.57E - 03	0.627	−0.58	−2.047	70
GOmf_oxidoreduction_driven_active_transmembrane_transporter_activity	1.31E - 05	3.82E - 03	0.593	−0.637	−2.049	42
HP_age_of_death	3.27E - 08	4.46E - 05	0.72	−0.441	−1.889	270
HP_abnormality_of_acid_base_homeostasis	1.65E - 07	1.92E - 04	0.69	−0.424	−1.826	282
HP_death_in_infancy	3.30E - 07	2.92E - 04	0.675	−0.495	−1.98	149
HP_hypertrophic_cardiomyopathy	3.58E - 07	2.92E - 04	0.675	−0.474	−1.928	169
HP_lactic_acidosis	8.25E - 07	5.18E - 04	0.659	−0.499	−1.969	132
HP_abnormal_celluIar_phenotype	1.28E - 06	6.96E - 04	0.644	−0.362	−1.614	451
HP_abnormal_liver_metabolite_concentration	2.73E - 06	1.31E - 03	0.627	−0.505	−1.945	114
HP_increased_serum_lactate	3.53E - 06	1.57E - 03	0.627	−0.461	−1.847	153
HP_atrophy_degeneration_affecting_the_cerebrum	5.02E - 06	1.95E - 03	0.611	−0.356	−1.582	439
HP_abnormal_enzyme_concentration_or_activity	5.65E - 06	2.10E - 03	0.611	−0.366	−1.621	401
HP_abnormality_of_the_mitochondrion	1.74E - 05	4.57E - 03	0.576	−0.449	−1.791	152
HP_lethargy	2.52E - 05	5.93E - 03	0.576	−0.473	−1.851	122
HP_abnormal_glucose_homeostasis	3.16E - 05	6.63E - 03	0.557	−0.362	−1.592	366
HP_abnormal_tissue_metabolite_concentration	4.01E - 05	7.91E - 03	0.557	−0.443	−1.777	154
HP_hemivertebrae	4.78E - 05	8.48E - 03	0.557	−0.646	−2.014	36
HP_encephalopathy	5.07E - 05	8.62E - 03	0.557	−0.425	−1.722	173
HP_hepatomegaly	6.18E - 05	9.32E - 03	0.538	−0.344	−1.528	420
HP_aplasia_hypoplasia_of_the_uterus	6.52E - 05	9.46E - 03	0.538	−0.64	−2.031	39

## Data Availability

The data used to support the findings of this study are available from the corresponding author upon reasonable request.
