# Supplementary material for: Transcriptomic Profiles and Functional Correlates of Cancer-Related Fatigue: A Cross-Sectional Study in Women Undergoing Cancer Treatment
Source: Eur J Cancer Care (Engl). Author manuscript; Available in PMC 2025 Aug 20. (PMC12362314; doi:10.1155/ecc/1092518)
Supplement: suppl — Supporting Figure 1: Calculation of the Static Fatigue Index. Supporting Figure 2: Heatmap correlating patient-reported fatigue, functional measures, and cell types as measured using clinical CBCs and RNA-Seq plus CIBERSORTx. Supporting Figure 3: PCA was used to identify clusters regarding demographics, clinical characteristics, or cell populations in blood. Supporting Figure 4: Raw data for an example single gene (SH3RF1) expression vs. fatigue. Supporting Table 1: Statistical alignment. Supporting Table 2: Differential Gene Expression for High vs Low Fatigue. Supporting Table 3: Differential Gene Expression for fatigue as a continuous variable. Supporting Table 4: Sensitivity analysis using the “leave one out” method. Supporting Table 5: Pathway analysis with fatigue as a continuous variable. [file NIHMS2103762-supplement-suppl.docx]

# Supplemental material

# Suppl. Fig. 1. Calculation of the Static Fatigue Index [1]. A higher static fatigue index indicates higher fatigability.


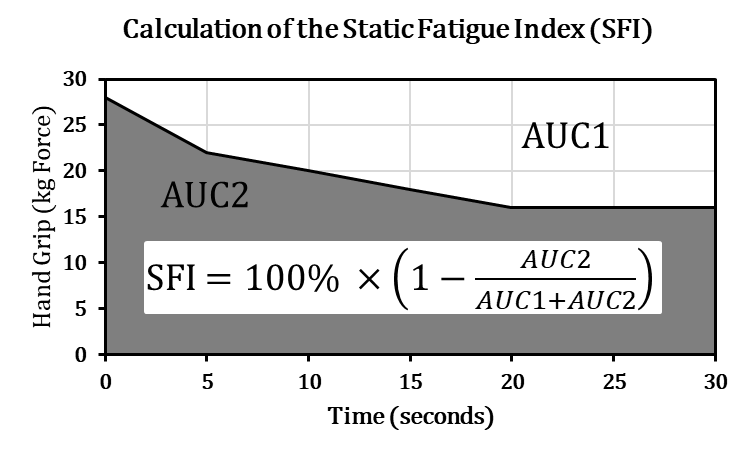


# Suppl. Fig. 2 Heat map correlating patient-reported fatigue, functional measures, and cell types as measured using clinical complete blood counts (CBCs) and RNA-Seq plus CIBERSORTx [2,3].

Key:

Level= fatigue at recruitment (not at blood collection.)

FACIT_Fatigue = Functional Assessment of Chronic Illness Therapy questionnaire- fatigue subscale

Age = age in years

BMI_kgm2 = body mass index in kg/m^2^

Stage = cancer stage

Albumin = albumin concentration from the clinical Complete Blood Count (CBC)

RBC = red blood cell concentration from the clinical Complete Blood Count (CBC)

WBC = white blood cell concentration from the clinical Complete Blood Count (CBC)

Platelets = platelet concentration from the clinical Complete Blood Count (CBC)

Neutrophils.x = neutrophil concentration from the clinical Complete Blood Count (CBC)

Monocytes.x = monocyte concentration from the clinical Complete Blood Count (CBC)

HandGripR_avg = hand grip strength on the right side, average of 3 trials

HandGripL_avg = hand grip strength on the left side, average of 3 trials

SFI_R_avg = static fatigue index on the right side

SFI_L_avg = static fatigue index on the left side

Static_Fatigue_Index_avg = static fatigue index, average of right hand and left hand trials

B.cells.naive = prediction of naïve B cell count from CIBERSORT

B.cells.memory = prediction of naïve B cell count from CIBERSORT

Plasma.cells = prediction of naïve B cell count from CIBERSORT

T.cells.CD8 = prediction of naïve B cell count from CIBERSORT

T.cells.CD4.naive = prediction of naïve B cell count from CIBERSORT

T.cells.CD4.memory.resting = prediction of resting memory CD4 T cell count from CIBERSORT

T.cells.CD4.memory.activated = prediction of activated memory CD4 T cell count from CIBERSORT

T.cells.regulatory_Tregs = prediction of regulatory T cell count from CIBERSORT

NK.cells.resting = prediction of resting natural killer cell count from CIBERSORT

NK.cells.activated = prediction of activated natural killer cell count from CIBERSORT

Monocytes.y = prediction of monocyte cell count from CIBERSORT

Macrophages.M0 = prediction of M0 macrophage cell count from CIBERSORT

Macrophages.M2 = prediction of M2 macrophage cell count from CIBERSORT Dendritic.cells.activated = prediction of activated dendtric cell count from CIBERSORT

Mast.cells.resting = prediction of resting mast cell count from CIBERSORT

Neutrophils.y = prediction of neutrophils cell count from CIBERSORT

# Suppl. Fig. 3. Principle Component Analysis was used to identify clusters regarding demographics, clinical characteristics, or cell populations in blood. These variables were used as covariates in the differentially expressed gene (DEG) analyses. Principle Component (PC1) accounted for 46% of the variance in transcript profile and PC2 accounted for 14% of the variance. For panel A, Cancer Stage, Stage 1 is orange, Stage 2 is green, and Stage 3 is blue. For panels B-F, each measure was dichotomized at the median; orange dots represent participants in the “high” group vs. the “low” group.


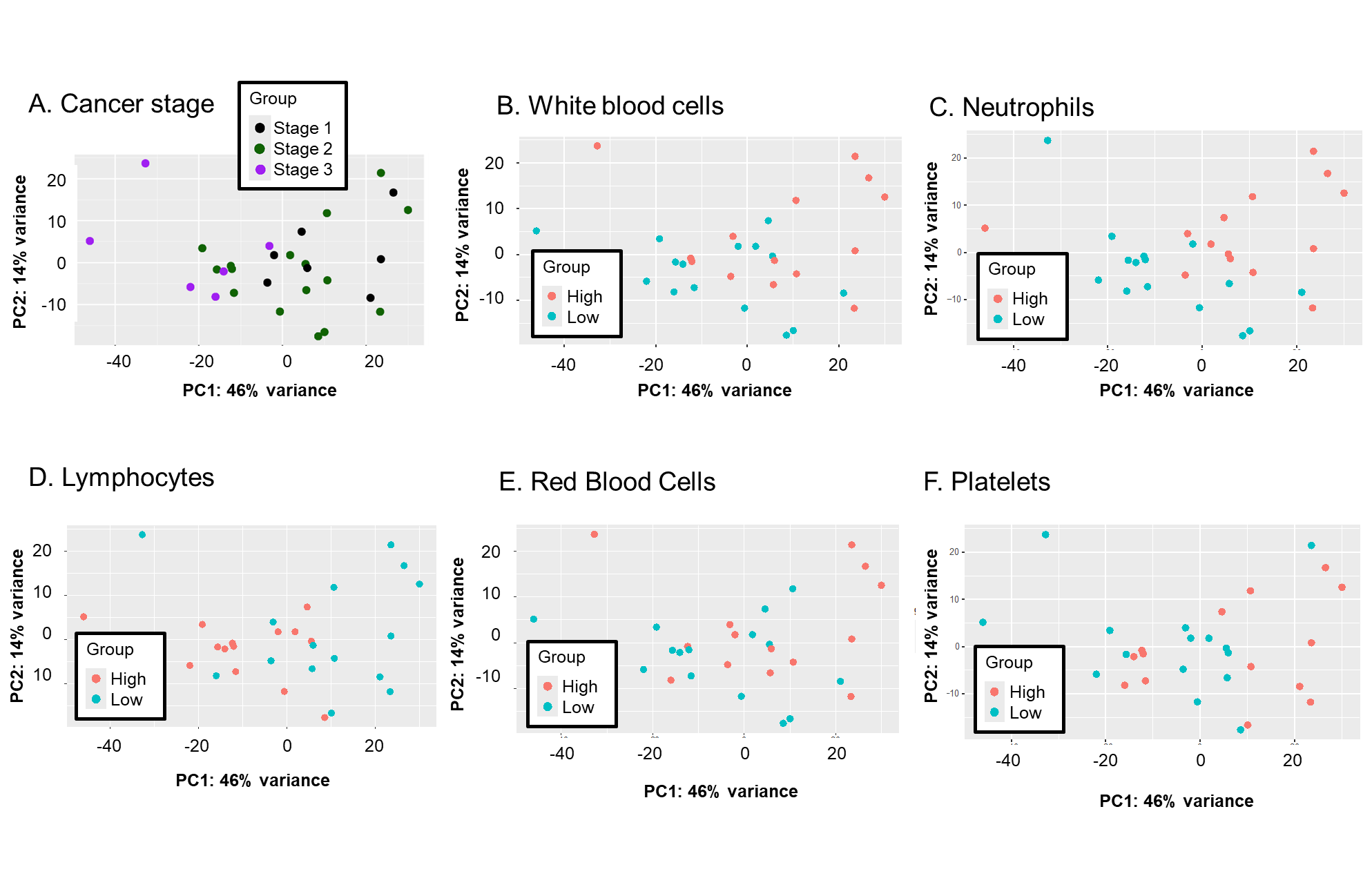


# Suppl. Fig. 4. Raw data for an example single gene (*SH3RF1*) expression vs. fatigue. A) Gene expression by fatigue with fatigue categorized as a binary variable. B) *SH3RF1* gene expression vs. fatigue as a continuous variable. As a continuous variable, a higher score on the FACIT-F is higher quality of life and less fatigue.


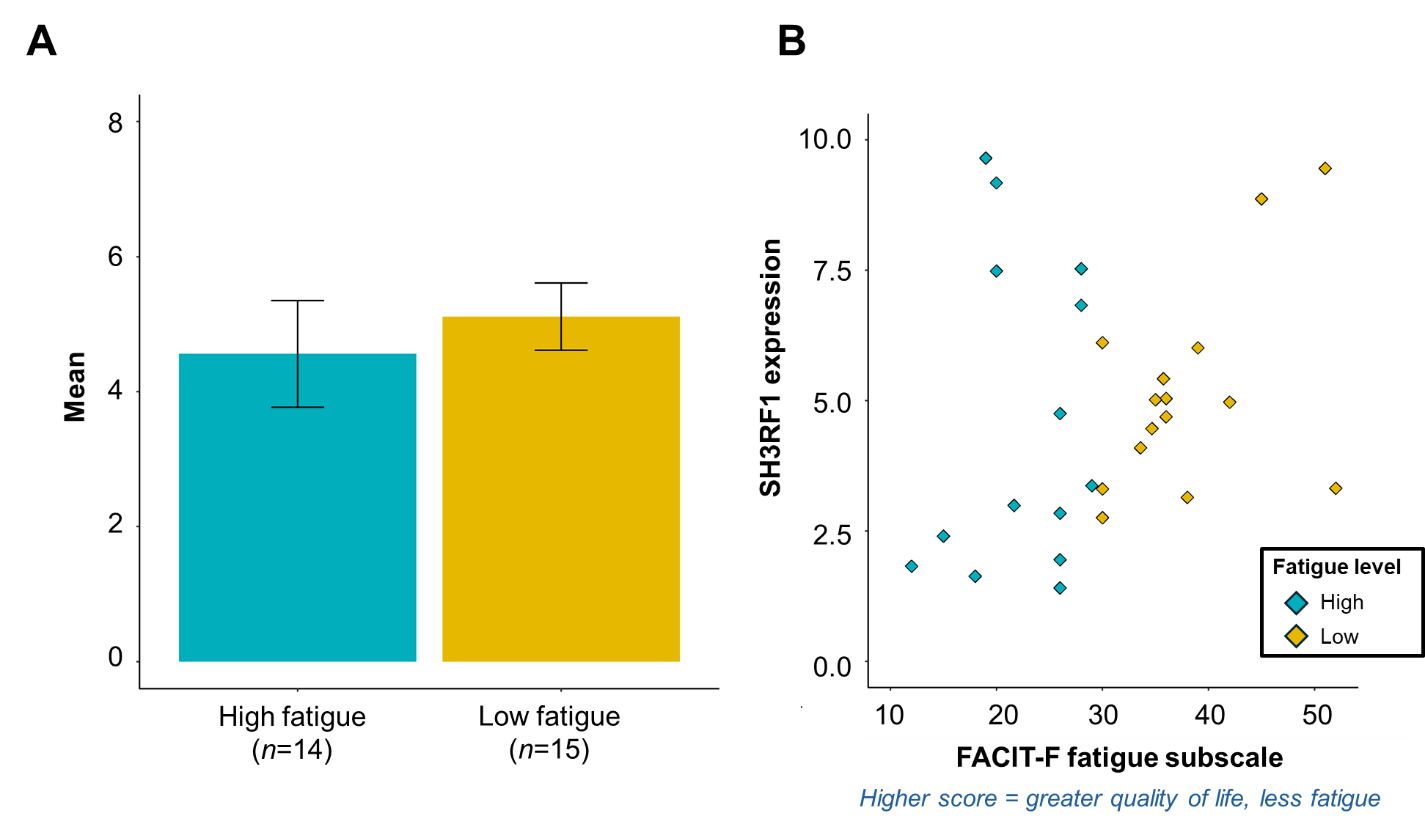


**Suppl. Table 1.** Statistical alignment

| **ID** | **Total reads** | **Mapped reads** | **Percent Mapped** | **Percent Properly Paired** | **Exon** | **Intron** | **Intergenic** |
| --- | --- | --- | --- | --- | --- | --- | --- |
| TRX01 | 94039106 | 90790667 | 96.55 | 96.59 | 91.22 | 7.60 | 1.18 |
| TRX02 | 90175188 | 87373862 | 96.89 | 96.56 | 92.24 | 6.69 | 1.07 |
| TRX03 | 96492680 | 93890838 | 97.30 | 97.06 | 96.68 | 2.81 | 0.51 |
| TRX04 | 110047872 | 106750165 | 97.00 | 96.57 | 95.47 | 3.84 | 0.69 |
| TRX05 | 94063454 | 91096720 | 96.85 | 96.87 | 95.00 | 4.16 | 0.84 |
| TRX06 | 122748564 | 117935583 | 96.08 | 96.11 | 89.16 | 9.39 | 1.45 |
| TRX07 | 115933548 | 111565729 | 96.23 | 96.03 | 90.56 | 8.03 | 1.42 |
| TRX08 | 115943636 | 111340292 | 96.03 | 96.04 | 91.07 | 7.69 | 1.24 |
| TRX09 | 119442650 | 115854079 | 97.00 | 96.84 | 94.06 | 5.02 | 0.91 |
| TRX10 | 120622180 | 116814443 | 96.84 | 96.81 | 92.80 | 6.24 | 0.97 |
| TRX11 | 101955220 | 98602969 | 96.71 | 96.61 | 89.72 | 8.96 | 1.32 |
| TRX12 | 86369620 | 84006970 | 97.26 | 97.06 | 95.28 | 4.04 | 0.68 |
| TRX13 | 99463578 | 95727736 | 96.24 | 96.10 | 91.05 | 7.77 | 1.19 |
| TRX14 | 141084834 | 137182262 | 97.23 | 97.17 | 97.05 | 2.51 | 0.44 |
| TRX15 | 121743026 | 118708250 | 97.51 | 97.00 | 96.51 | 2.93 | 0.56 |
| TRX16 | 139290742 | 135350009 | 97.17 | 97.02 | 95.57 | 3.73 | 0.70 |
| TRX17 | 102362116 | 97731341 | 95.48 | 95.47 | 86.89 | 11.18 | 1.94 |
| TRX18 | 133466472 | 129872023 | 97.31 | 97.00 | 95.14 | 4.17 | 0.69 |
| TRX19 | 100749726 | 97499704 | 96.77 | 96.58 | 93.78 | 5.32 | 0.90 |
| TRX20 | 109178206 | 106151702 | 97.23 | 97.03 | 96.00 | 3.42 | 0.58 |
| TRX21 | 106971200 | 103819138 | 97.05 | 96.79 | 95.61 | 3.77 | 0.62 |
| TRX22 | 110197670 | 106281131 | 96.45 | 96.47 | 92.56 | 6.47 | 0.97 |
| TRX23 | 102726406 | 99684179 | 97.04 | 96.74 | 93.41 | 5.70 | 0.89 |
| TRX24 | 118728450 | 114667547 | 96.58 | 96.58 | 92.28 | 6.60 | 1.12 |
| TRX25 | 99075944 | 95272206 | 96.16 | 95.85 | 85.24 | 12.35 | 2.40 |
| TRX26 | 30143646 | 29201368 | 96.87 | 96.93 | 96.35 | 3.07 | 0.58 |
| TRX27 | 101601436 | 98421740 | 96.87 | 96.77 | 92.92 | 6.08 | 1.00 |
| TRX28 | 98718948 | 95530687 | 96.77 | 96.64 | 93.63 | 5.52 | 0.85 |
| TRX29 | 106057806 | 102978605 | 97.10 | 96.78 | 94.40 | 4.82 | 0.79 |
| TRX30 | 100859574 | 97582928 | 96.75 | 96.62 | 90.48 | 8.29 | 1.24 |
|  |  |  |  |  |  |  |  |
| Average | 106341783 | 102922829 | 96.78 | 96.62 | 93.07 | 5.94 | 0.99 |

**Suppl. Table 2.** Differential Gene Expression for High vs Low Fatigue (first 20 rows)

| **Gene** | **Base Mean** | **Linear Fold Change** | **Log2 Fold Change** | **Log Fold Change SE** | **Statistic** | ***p*-value** | **Adjusted *p*-value** |
| --- | --- | --- | --- | --- | --- | --- | --- |
| *SH3RF1* | 88.07 | -2.06 | -1.04 | 0.20 | 26.44 | 2.72E-07 | 4.08E-03 |
| *CAPRIN2* | 519.54 | 1.34 | 0.42 | 0.09 | 23.75 | 1.10E-06 | 8.24E-03 |
| *BIRC2* | 3130.77 | 1.96 | 0.97 | 0.23 | 20.90 | 4.84E-06 | 2.42E-02 |
| *CLEC4C* | 79.27 | 2.85 | 1.51 | 0.37 | 15.61 | 7.79E-05 | 1.97E-01 |
| *ZNF731P* | 778.03 | 1.38 | 0.46 | 0.12 | 15.58 | 7.89E-05 | 1.97E-01 |
| *HMOX2* | 700.79 | -1.24 | -0.31 | 0.08 | 14.92 | 1.12E-04 | 2.00E-01 |
| *FBP1* | 307.72 | -1.50 | -0.59 | 0.15 | 14.57 | 1.35E-04 | 2.00E-01 |
| *PPP2R1A* | 1758.37 | -1.27 | -0.34 | 0.09 | 14.46 | 1.43E-04 | 2.00E-01 |
| *SLC25A5* | 1772.59 | -1.26 | -0.33 | 0.09 | 14.37 | 1.50E-04 | 2.00E-01 |
| *COX5A* | 488.92 | -1.34 | -0.42 | 0.11 | 14.25 | 1.60E-04 | 2.00E-01 |
| *MYDGF* | 345.62 | -1.31 | -0.40 | 0.11 | 13.64 | 2.21E-04 | 2.37E-01 |
| *PRPF31* | 597.97 | -1.25 | -0.33 | 0.09 | 13.58 | 2.28E-04 | 2.37E-01 |
| *SLBP* | 908.68 | -1.22 | -0.28 | 0.08 | 13.47 | 2.42E-04 | 2.37E-01 |
| *CLIC2* | 520.28 | 2.24 | 1.17 | 0.35 | 13.30 | 2.65E-04 | 2.37E-01 |
| *SMIM29* | 206.54 | -1.32 | -0.40 | 0.11 | 13.27 | 2.69E-04 | 2.37E-01 |
| *POLI* | 524.44 | 1.30 | 0.38 | 0.11 | 12.91 | 3.26E-04 | 2.61E-01 |
| *YOD1* | 2525.95 | 2.10 | 1.07 | 0.33 | 12.89 | 3.31E-04 | 2.61E-01 |
| *EPS8* | 79.17 | -1.70 | -0.77 | 0.21 | 12.72 | 3.63E-04 | 2.61E-01 |
| *CCNL1* | 5081.50 | 1.30 | 0.37 | 0.11 | 12.66 | 3.73E-04 | 2.61E-01 |
| *SNRPG* | 216.57 | -1.51 | -0.60 | 0.16 | 12.57 | 3.92E-04 | 2.61E-01 |

**Suppl. Table 3.** Differential Gene Expression for fatigue as a continuous variable. A higher score on the FACIT-F indicates higher quality life and less fatigue.

| **Gene** | **Base Mean** | **Linear Fold Change** | **Log2 Fold Change** | **Log Fold Change SE** | **Statistic** | ***p*-value** | **Adjusted *p*-value** |
| --- | --- | --- | --- | --- | --- | --- | --- |
| *SH3RF1* | 88.07 | 1.43 | 0.52 | 0.08 | 42.95 | 5.61E-11 | 5.64E-07 |
| *CAPRIN2* | 519.54 | -1.15 | -0.20 | 0.04 | 32.57 | 1.15E-08 | 5.77E-05 |
| *CS* | 2244.37 | 1.07 | 0.10 | 0.02 | 26.41 | 2.76E-07 | 9.26E-04 |
| *FAM153A* | 414.80 | -1.60 | -0.68 | 0.13 | 23.06 | 1.57E-06 | 3.95E-03 |
| *MRPL51* | 414.75 | 1.17 | 0.22 | 0.05 | 18.84 | 1.42E-05 | 2.79E-02 |
| *MAPKAPK3* | 2116.57 | 1.11 | 0.15 | 0.03 | 18.54 | 1.67E-05 | 2.79E-02 |
| *VPS18* | 674.54 | 1.10 | 0.14 | 0.03 | 17.38 | 3.07E-05 | 4.41E-02 |
| *ZNF731P* | 778.03 | -1.16 | -0.21 | 0.05 | 16.59 | 4.64E-05 | 4.82E-02 |
| *PPP2R1A* | 1758.37 | 1.12 | 0.16 | 0.04 | 16.41 | 5.10E-05 | 4.82E-02 |
| *CHCHD2* | 1423.57 | 1.13 | 0.18 | 0.04 | 16.30 | 5.42E-05 | 4.82E-02 |
| *SMIM29* | 206.54 | 1.15 | 0.20 | 0.05 | 16.20 | 5.70E-05 | 4.82E-02 |
| *SLC25A3* | 3289.96 | 1.05 | 0.07 | 0.02 | 16.18 | 5.75E-05 | 4.82E-02 |
| *PRPF39* | 647.77 | -1.10 | -0.14 | 0.04 | 15.78 | 7.12E-05 | 5.40E-02 |
| *ZNF506* | 712.64 | -1.16 | -0.21 | 0.05 | 15.60 | 7.83E-05 | 5.40E-02 |
| *HIRA* | 231.02 | 1.14 | 0.19 | 0.05 | 15.55 | 8.06E-05 | 5.40E-02 |
| *GIMAP5* | 478.52 | -1.21 | -0.28 | 0.07 | 15.16 | 9.87E-05 | 6.20E-02 |
| *ZNF701* | 342.18 | -1.13 | -0.18 | 0.05 | 14.98 | 1.08E-04 | 6.33E-02 |
| *STEEP1* | 288.16 | 1.14 | 0.18 | 0.05 | 14.90 | 1.14E-04 | 6.33E-02 |
| *HMOX2* | 700.79 | 1.10 | 0.14 | 0.04 | 14.72 | 1.24E-04 | 6.33E-02 |
| *ARHGDIA* | 3889.09 | 1.13 | 0.17 | 0.04 | 14.65 | 1.29E-04 | 6.33E-02 |

**Suppl. Table 4.** Sensitivity analysis using the “leave one out” method. There were a total of 29 genes, so the range for the last column is 0-28.

| **Gene** | **Regulation in high vs. low fatigue (binary variable)** | **Times it is significant** |
| --- | --- | --- |
| *CAPRIN2* | up | 28 |
| *BIRC2* | up | 27 |
| *ZNF731P* | up | 27 |
| *SH3RF1* | down | 26 |
| *CLEC4C* | up | 25 |
| *SLC25A5* | down | 25 |
| *FBP1* | down | 24 |
| *HMOX2* | down | 24 |
| *PPP2R1A* | down | 24 |
| *COX5A* | down | 23 |
| *CLIC2* | up | 22 |
| *MYDGF* | down | 22 |
| *PRPF31* | down | 22 |
| *SLBP* | down | 19 |
| *SMIM29* | down | 17 |
| *YOD1* | up | 16 |
| *POLI* | up | 15 |
| *SLC1A3* | up | 15 |
| *CCNL1* | up | 13 |
| *EPS8* | down | 13 |
| *FAM153A* | up | 13 |
| *HCG27* | up | 12 |
| *SEC62* | up | 10 |
| *SNRPG* | down | 8 |
| *CUBN* | up | 7 |
| *VAMP8* | down | 7 |
| *NACC1* | down | 5 |
| *PFDN1* | down | 5 |
| *PRPF6* | down | 5 |
| *ARHGDIA* | down | 4 |

# Suppl. Table 5. Pathway analysis with fatigue as a continuous variable. A higher score on the Functional Assessment of Chronic Illness-Fatigue (FACIT-F) fatigue subscale indicates higher quality of life and less fatigue.

| **Pathway** | ***p*-value** | **Adjusted *p*-value** | **log2 error** | **Effect Size** | **Normalized Effect Size** | **Size** |
| --- | --- | --- | --- | --- | --- | --- |
| GOBP_GENERATION_OF_PRECURSOR_METABOLITES_AND_ENERGY | 2.51E-12 | 2.05E-08 | 0.89867123 | 0.47593462 | 2.02313618 | 315 |
| GOBP_AEROBIC_RESPIRATION | 1.90E-10 | 5.18E-07 | 0.8266573 | 0.57391598 | 2.22127111 | 131 |
| GOBP_CELLULAR_RESPIRATION | 1.39E-09 | 2.84E-06 | 0.78818681 | 0.53880305 | 2.11675158 | 154 |
| GOBP_ENERGY_DERIVATION_BY_OXIDATION_OF_ORGANIC_COMPOUNDS | 3.90E-08 | 4.55E-05 | 0.71951283 | 0.48022434 | 1.95409064 | 201 |
| GOBP_OXIDATIVE_PHOSPHORYLATION | 8.62E-08 | 8.80E-05 | 0.70497572 | 0.57897203 | 2.15089873 | 95 |
| GOBP_PROTON_TRANSMEMBRANE_TRANSPORT | 1.76E-06 | 9.00E-04 | 0.64355184 | 0.57813186 | 2.08111081 | 78 |
| GOBP_RESPIRATORY_ELECTRON_TRANSPORT_CHAIN | 2.93E-06 | 0.00132851 | 0.62725674 | 0.56266938 | 2.01688888 | 77 |
| GOBP_CARBOHYDRATE_DERIVATIVE_BIOSYNTHETIC_PROCESS | 3.67E-06 | 0.00144546 | 0.62725674 | 0.3719709 | 1.61893616 | 392 |
| GOBP_ELECTRON_TRANSPORT_CHAIN | 3.66E-06 | 0.00144546 | 0.62725674 | 0.51023719 | 1.94062342 | 112 |
| GOBP_ATP_SYNTHESIS_COUPLED_ELECTRON_TRANSPORT | 4.71E-06 | 0.00168047 | 0.61052688 | 0.59306157 | 2.07450482 | 66 |
| GOBP_CHROMOSOME_ORGANIZATION | 7.09E-06 | 0.00231524 | 0.61052688 | 0.3633226 | 1.58273342 | 412 |
| GOBP_PROTON_MOTIVE_FORCE_DRIVEN_ATP_SYNTHESIS | 9.61E-06 | 0.00290603 | 0.59332548 | 0.6190586 | 2.06355885 | 52 |
| GOBP_RIBOSE_PHOSPHATE_BIOSYNTHETIC_PROCESS | 1.04E-05 | 0.00296394 | 0.59332548 | 0.46128198 | 1.82072159 | 156 |
| GOBP_NUCLEOSIDE_PHOSPHATE_BIOSYNTHETIC_PROCESS | 1.64E-05 | 0.00393876 | 0.57561026 | 0.42944307 | 1.73926994 | 191 |
| GOBP_SPLICEOSOMAL_SNRNP_ASSEMBLY | 2.14E-05 | 0.00438144 | 0.57561026 | 0.69441802 | 2.1146363 | 33 |
| GOBP_U2_TYPE_PRESPLICEOSOME_ASSEMBLY | 2.04E-05 | 0.00438144 | 0.57561026 | 0.76903305 | 2.09962677 | 22 |
| GOBP_RIBONUCLEOPROTEIN_COMPLEX_BIOGENESIS | 2.77E-05 | 0.00513754 | 0.57561026 | 0.36231381 | 1.5622001 | 371 |
| GOBP_RNA_SPLICING_VIA_TRANSESTERIFICATION_REACTIONS | 3.81E-05 | 0.00662357 | 0.55733224 | 0.39430217 | 1.65190035 | 247 |
| GOBP_ATP_BIOSYNTHETIC_PROCESS | 5.73E-05 | 0.00865883 | 0.55733224 | 0.54607755 | 1.92115625 | 68 |
| GOBP_NUCLEOBASE_CONTAINING_SMALL_MOLECULE_METABOLIC_PROCESS | 5.46E-05 | 0.00865883 | 0.55733224 | 0.35758855 | 1.54179117 | 365 |
| GOCC_MITOCHONDRIAL_PROTEIN_CONTAINING_COMPLEX | 5.34E-11 | 2.18E-07 | 0.85133906 | 0.52180595 | 2.12467259 | 202 |
| GOCC_INNER_MITOCHONDRIAL_MEMBRANE_PROTEIN_COMPLEX | 6.93E-09 | 1.13E-05 | 0.7614608 | 0.58734204 | 2.20036412 | 103 |
| GOCC_ORGANELLE_INNER_MEMBRANE | 2.07E-07 | 1.88E-04 | 0.69013246 | 0.39994059 | 1.72195428 | 349 |
| GOCC_ATPASE_COMPLEX | 5.59E-07 | 4.15E-04 | 0.6594444 | 0.53897572 | 2.0347068 | 109 |
| GOCC_ENDOPLASMIC_RETICULUM_PROTEIN_CONTAINING_COMPLEX | 6.82E-07 | 4.64E-04 | 0.6594444 | 0.54797216 | 2.03991473 | 98 |
| GOCC_SM_LIKE_PROTEIN_FAMILY_COMPLEX | 1.01E-06 | 6.33E-04 | 0.64355184 | 0.58804572 | 2.09981471 | 73 |
| GOCC_RESPIRASOME | 2.22E-06 | 0.00106731 | 0.62725674 | 0.60333745 | 2.09793085 | 63 |
| GOCC_PRECATALYTIC_SPLICEOSOME | 7.06E-06 | 0.00231524 | 0.61052688 | 0.64943918 | 2.10580377 | 46 |
| GOCC_FICOLIN_1_RICH_GRANULE_LUMEN | 7.65E-06 | 0.00240371 | 0.59332548 | 0.5118879 | 1.91429859 | 102 |
| GOCC_ENDOPEPTIDASE_COMPLEX | 1.05E-05 | 0.00296394 | 0.59332548 | 0.58515412 | 2.04729368 | 67 |
| GOCC_SPLICEOSOMAL_SNRNP_COMPLEX | 1.49E-05 | 0.00379615 | 0.59332548 | 0.59813646 | 2.02853115 | 57 |
| GOCC_PEPTIDASE_COMPLEX | 1.57E-05 | 0.00387786 | 0.57561026 | 0.52028272 | 1.92439747 | 92 |
| GOCC_SPLICEOSOMAL_COMPLEX | 2.20E-05 | 0.00438144 | 0.57561026 | 0.43814846 | 1.74591583 | 172 |
| GOCC_U2_SNRNP | 2.20E-05 | 0.00438144 | 0.57561026 | 0.7507022 | 2.07714271 | 23 |
| GOCC_U2_TYPE_CATALYTIC_STEP_2_SPLICEOSOME | 2.06E-05 | 0.00438144 | 0.57561026 | 0.71167056 | 2.09639356 | 28 |
| GOCC_U2_TYPE_SPLICEOSOMAL_COMPLEX | 2.00E-05 | 0.00438144 | 0.57561026 | 0.52832009 | 1.930165 | 83 |
| GOCC_OXIDOREDUCTASE_COMPLEX | 3.53E-05 | 0.00625991 | 0.55733224 | 0.52269862 | 1.91257969 | 84 |
| GOCC_MITOCHONDRIAL_MATRIX | 4.04E-05 | 0.00686646 | 0.55733224 | 0.3687896 | 1.58326394 | 345 |
| GOCC_FICOLIN_1_RICH_GRANULE | 6.40E-05 | 0.00917342 | 0.5384341 | 0.44984118 | 1.76382536 | 146 |
| GOMF_OXIDOREDUCTASE_ACTIVITY | 2.59E-08 | 3.53E-05 | 0.73376199 | 0.40214255 | 1.75007231 | 389 |
| GOMF_PROTON_TRANSMEMBRANE_TRANSPORTER_ACTIVITY | 2.38E-05 | 0.00463682 | 0.57561026 | 0.5624309 | 1.99507596 | 70 |
| GOMF_NUCLEOSOME_BINDING | 2.59E-05 | 0.00491051 | 0.57561026 | 0.61724444 | 2.02989615 | 50 |
| GOMF_ELECTRON_TRANSFER_ACTIVITY | 5.83E-05 | 0.00865883 | 0.55733224 | 0.5437349 | 1.94582754 | 74 |
| GOMF_OXIDOREDUCTION_DRIVEN_ACTIVE_TRANSMEMBRANE_  TRANSPORTER_ACTIVITY | 6.37E-05 | 0.00917342 | 0.5384341 | 0.63226103 | 2.02158039 | 42 |
| HP_LACTIC_ACIDOSIS | 4.69E-07 | 3.83E-04 | 0.67496286 | 0.50824152 | 1.96795807 | 132 |
| HP_ABNORMALITY_OF_ACID_BASE_HOMEOSTASIS | 1.29E-06 | 7.53E-04 | 0.64355184 | 0.41697161 | 1.76467772 | 282 |
| HP_ABNORMAL_LIVER_METABOLITE_CONCENTRATION | 1.70E-06 | 9.00E-04 | 0.64355184 | 0.50939345 | 1.94004218 | 114 |
| HP_AGE_OF_DEATH | 3.72E-06 | 0.00144546 | 0.62725674 | 0.40767289 | 1.71908551 | 270 |
| HP_ABNORMAL_ENZYME_CONCENTRATION_OR_ACTIVITY | 4.73E-06 | 0.00168047 | 0.61052688 | 0.37314072 | 1.62882937 | 401 |
| HP_ABNORMAL_CELLULAR_PHENOTYPE | 1.20E-05 | 0.00326501 | 0.59332548 | 0.35258916 | 1.55455829 | 451 |
| HP_ABNORMALITY_OF_THE_MITOCHONDRION | 1.45E-05 | 0.00379615 | 0.59332548 | 0.45333766 | 1.77608678 | 152 |
| HP_INCREASED_SERUM_LACTATE | 1.85E-05 | 0.00430615 | 0.57561026 | 0.44895713 | 1.76697934 | 153 |
| HP_DEATH_IN_INFANCY | 2.99E-05 | 0.00543236 | 0.57561026 | 0.44731356 | 1.75614406 | 149 |
| HP_ENCEPHALOPATHY | 4.74E-05 | 0.00790445 | 0.55733224 | 0.42006768 | 1.67234994 | 173 |
| HP_ATROPHY_DEGENERATION_AFFECTING_THE_CEREBRUM | 4.94E-05 | 0.00806676 | 0.55733224 | 0.34334591 | 1.50525715 | 439 |
| HP_HEPATOMEGALY | 5.65E-05 | 0.00865883 | 0.55733224 | 0.34158831 | 1.49243551 | 420 |
| HP_HYPERTROPHIC_CARDIOMYOPATHY | 5.79E-05 | 0.00865883 | 0.55733224 | 0.43642727 | 1.73565482 | 169 |

# References

1. Feng LR, Regan J, Shrader JA, Liwang J, Ross A, Kumar S*, et al.* Cognitive and motor aspects of cancer-related fatigue. Cancer Med **2019**;8:5840-9

2. Chen B, Khodadoust MS, Liu CL, Newman AM, Alizadeh AA. Profiling Tumor Infiltrating Immune Cells with CIBERSORT. Methods in Molecular Biology. Totowa, NJ, USA: Humana Press Inc; 2018. p 243-59.

3. Steen CB, Liu CL, Alizadeh AA, Newman AM. Profiling Cell Type Abundance and Expression in Bulk Tissues with CIBERSORTx. Methods in Molecular Biology. Milwaukee, WI, USA: NIH Public Access; 2020. p 135-57.
